# Direction-Dependent Applicability of Grey Forecasting Models for Spatiotemporal Chloride Concentration Prediction in Concrete

**DOI:** 10.3390/ma19214484

**Published:** 2026-10-24

**Authors:** Lurong Hua, Yuquan Hu, Xingqi Xu, Liunian Shi

**Affiliations:** 1 School of Urban Construction, Jiangsu Open University, Nanjing 210019, China; lorraine42@163.com; 2 School of Water Conservancy and Transportation, Zhengzhou University, Zhengzhou 450001, China; 18358333489@163.com (X.X.); 19803813827@163.com (L.S.)

**Keywords:** chloride concentration prediction, grey forecasting model, temporal prediction, spatial prediction, concrete durability

## Abstract

Accurately predicting chloride concentration is crucial for the durability assessment of reinforced concrete structures. This study evaluates the direction-dependent applicability of four grey forecasting models, including conventional GM (1,1), Markov residual-corrected GM (1,1), GM (1,1) with full order time power terms (FOTP-GM (1,1)), and improved metabolic GM (1,1), for temporal and spatial chloride concentration prediction. Temporal sequences were constructed at fixed depths of 10 and 14 mm, while spatial sequences were obtained at exposure times of 280 and 320 d. Holdout, rolling one-step, and rolling two-step forecasting were employed, and model performance was assessed using statistical accuracy, numerical stability, and physical admissibility. The results showed that the temporal sequences exhibited quasi-linear growth, whereas the spatial profiles showed approximately exponential-like attenuation. Among the four grey models, FOTP-GM showed the most consistent temporal forecasting performance, with holdout MAPEs of only 2.1–2.3% and a pooled rolling one-step WAPE of 7.49%. Comparison with reference benchmarks further showed that simple linear and Fick-based models remained competitive for temporal forecasting, while the Fick-based model provided the highest accuracy for spatial extrapolation. In contrast, no single model was consistently superior for spatial prediction; although Markov-GM and FOTP-GM reduced overall statistical errors, both produced nonphysical negative concentrations in the deep low-concentration region. The optimal FOTP order was also generally higher for temporal sequences than for spatial sequences, further demonstrating the dependence of model complexity on forecasting direction. These findings reveal that under the investigated 28-d curing and exposure conditions, the grey forecasting models exhibited clear direction-dependent applicability associated with the distinct characteristics of temporal and spatial chloride sequences, owing to the distinct sequence characteristics of temporal accumulation and spatial attenuation. The proposed framework provides a rational basis for grey-model selection in spatiotemporal chloride concentration prediction and offers a practical small-sample approach for concrete durability assessment.

## 1. Introduction

The corrosion of steel reinforcement caused by chloride ions is one of the primary degradation factors affecting the durability and service life of reinforced concrete structures exposed to marine environments, deicing salts, and other chloride-containing media [1,2]. Chloride ions gradually penetrate through the concrete cover and accumulate around the reinforcement. Once the chloride concentration reaches a critical level, the passive film on the steel surface may become unstable and corrosion can be initiated. Therefore, reliable characterization and prediction of chloride ingress are crucial for evaluating corrosion risk and supporting the durability and service life design of reinforced concrete structures.

Chloride transport in concrete is a typical spatiotemporal process. Fick’s second law and its modified forms remain the most widely used theoretical approaches for describing chloride ingress [3,4]. However, chloride transport is influenced by multiple material and environmental factors. Cracking is another important factor affecting chloride transport, as cracks can provide preferential pathways for chloride ingress. Fiber reinforcement can mitigate this effect through crack bridging and crack-width control, although its effectiveness depends on fiber geometry, such as shape and aspect ratio, as well as fiber volume fraction [5]. The key parameters such as the apparent diffusion coefficient and surface chloride concentration may vary with exposure time, curing condition, and penetration depth [6,7]. Previous studies have shown that the diffusion coefficient can evolve during exposure, while the surface chloride concentration may progressively increase before approaching a relatively stable level [8,9]. Consequently, physically based chloride transport models require appropriate determination of boundary conditions and transport parameters, which often depends on relatively long-term experimental observations.

Long-duration chloride exposure tests are time-consuming and costly, and the number of available observations is therefore often limited. Grey forecasting theory provides a potentially useful complementary data-driven approach for such small-sample problems. Since its introduction for systems with incomplete and limited information, GM (1,1) has become one of the most widely used univariate grey forecasting models because it can establish a prediction relationship from a relatively small number of observations without requiring a large statistical sample [10]. For instance, in the field of concrete performance prediction, the GM (1,1) model is commonly utilized for predicting concrete compressive strength and evaluating frost resistance, offering a novel approach for predicting the durability lifespan of concrete [11]. Nevertheless, conventional GM (1,1) has an inherent exponential-type response structure, and its forecasting performance may deteriorate when the original sequence deviates substantially from a quasi-exponential pattern. Various improved grey models have therefore been developed to enhance its adaptability. Markov residual-corrected GM (1,1) retains the basic GM trend and uses residual information together with Markov state-transition probabilities to compensate for local prediction deviations [12]. FOTP-GM (1,1) introduces full-order power terms into the grey action function to improve the representation of non-homogeneous and non-exponential sequences [13]. Improved metabolic GM (1,1), in contrast, dynamically updates the modeling sequence by removing old information and incorporating newly generated information, thereby improving recursive forecasting capability [14]. These models represent three different improvement strategies; namely, residual correction, structural modification, and dynamic information updating.

Most previous applications of grey forecasting models have focused on temporally ordered data. For chloride transport, however, the same experimental concentration field can be represented in two different ways. At a fixed depth, chloride concentration evolves with exposure time, whereas at a fixed exposure age, chloride concentration decreases with penetration depth. These two sequence types originate from the same physical transport process but may exhibit substantially different mathematical characteristics. Previous chloride transport studies have shown that temporal concentration evolution is affected by the time dependence of transport properties and boundary conditions, while spatial concentration profiles generally exhibit pronounced monotonic attenuation [15,16]. Whether grey forecasting models developed primarily for temporal sequences can be directly extended to spatial chloride profiles, and whether the same model structure remains optimal in both directions, have not yet been systematically clarified. Another issue concerns the criteria used to evaluate model applicability. Most forecasting studies rank models primarily according to statistical error indices. For spatial chloride profiles, however, this approach may be insufficient because the chloride concentration decreases rapidly toward the interior of concrete. In the deep region, a small absolute prediction deviation may produce a very large percentage error. More importantly, residual correction or high-order extrapolation may generate negative chloride concentrations, which are mathematically possible but physically inadmissible. Therefore, the evaluation of grey forecasting models for chloride transport should consider not only statistical accuracy, but also model complexity, numerical stability, forecasting horizon, and physical reliability.

Accordingly, this study investigates the direction-dependent applicability of grey forecasting models for chloride concentration prediction. Four representative models, including conventional GM (1,1), Markov residual corrected GM (1,1), FOTP-GM (1,1), and improved metabolic GM (1,1), were evaluated for both temporal and spatial chloride concentration sequences using a unified framework comprising holdout validation, rolling one-step forecasting, and rolling two-step forecasting. Particular attention was paid to the relationships between sequence characteristics, model complexity, forecasting accuracy, numerical stability, and physical admissibility. By systematically comparing model performance in the temporal and spatial directions, this study examines how sequence structure is associated with the applicability and optimal complexity of grey forecasting models under the investigated conditions. Linear regression, exponential regression, and a classical Fick-based diffusion model were additionally included as reference benchmarks and evaluated using the same validation framework as the grey models.

## 2. Experimental Database and Sequence Construction

### 2.1. Experimental Database

The data used in this study were obtained from our previous experimental investigation on chloride ingress in steam-cured concrete, the results of which have been reported in Reference [9]. The experimental materials, specimen preparation, exposure conditions, equipment, and chloride measurement procedures, including their detailed specifications, were reported in the original study [9]. Briefly, chloride profiles were obtained by depth-wise powder sampling, and the free chloride content was determined according to JGJ/T 322-2013 [17]. In that study, steam-cured concrete specimens with curing ages of 3, 7, 14, and 28 d were exposed to a 3.5% NaCl solution for 40–320 d. Chloride concentration profiles were measured at different exposure ages and depths ranging from 1 to 50 mm, providing a spatiotemporal dataset of chloride ingress. The chloride concentration *C* reported in this study represents the water-soluble (free) chloride content expressed as a percentage by mass of mortar, in accordance with JGJ/T 322-2013. Because the present study reuses the published chloride-profile values as fixed modeling sequences, replicate-level variability and an instrument-specific analytical detection limit were not re-evaluated in the present analysis.

Grey forecasting models are conventionally developed for ordered temporal sequences. Mathematically, however, grey modeling operates on an ordered sequence rather than explicitly on physical time, allowing its extension to equally spaced spatial observations. Accordingly, the chloride field *C*(*x*, *t*) was decomposed into temporal and spatial sequences to investigate the direction-dependent applicability of the four grey forecasting models. For the temporal direction, the 28 d cured specimens at depths of 10 and 14 mm were selected to construct chloride concentration–time sequences. For the spatial direction, the chloride profiles of the 28 d cured specimens after 280 and 320 d of exposure were selected. The 6–42 mm interval was selected because it provides a uniformly spaced depth sequence with Δx=4. The near-surface points at 1 and 3 mm were excluded because of their different sampling intervals, while deeper zero-valued observations were excluded because the conventional grey-model formulation requires a strictly positive input sequence. The minimum nonzero concentration used in the analysis was 0.001%; this value represents the reported experimental concentration rather than an assumed analytical detection limit. The 28-d curing condition was selected as a representative mature curing condition to provide a consistent basis for comparing temporal and spatial forecasting behavior. The present analysis therefore focuses on model applicability under this specific curing condition rather than on the influence of curing age. The four selected datasets are summarized in Table 1.

### 2.2. Construction of Temporal and Spatial Sequences

For the temporal direction, the chloride concentration at a fixed depth *x* = *x_j_* was expressed as Equation (1).

(1) $$X_{T}^{\left(0\right)}=\left\{C(x_{j},t_{1}),C(x_{j},t_{2}),\ldots ,C(x_{j},t_{n})\right\}$$

where the exposure times were equally spaced at *t_k_* = 40 *k*, *k* = 1, 2, …, 8, corresponding to an interval of Δt=40 d. Two representative temporal sequences at depths of 10 and 14 mm were considered, each containing 8 data points.

For the spatial direction, the chloride concentration profile at a fixed exposure time *t* = *t_j_* was expressed as Equation (2).

(2) $$X_{S}^{\left(0\right)}=\left\{C(x_{1},t_{j}),C(x_{2},t_{j}),\ldots ,C(x_{n},t_{j})\right\}$$

where the selected depths ranged from 6 to 42 mm with a constant spacing of Δx=4 mm. Spatial sequences corresponding to exposure times of 280 and 320 d were selected, each containing 10 data points.

## 3. Grey Forecasting Models and Evaluation Framework

### 3.1. Conventional GM (1,1) Model

The conventional GM (1,1) model was adopted as the baseline grey forecasting model. For an original non-negative sequence $X^{\left(0\right)}=\left\{x^{\left(0\right)}(1),x^{\left(0\right)}(2),\ldots ,x^{\left(0\right)}(n)\right\}$, calculate the first-order accumulated sequence $X^{\left(1\right)}=\left\{x^{\left(1\right)}(1),x^{\left(1\right)}(2),\ldots ,x^{\left(1\right)}(n)\right\}$ according to Equation (3).

(3) $$x^{\left(1\right)}(k)=\sum_{i=1}^{k}x^{\left(0\right)}(i),\;\;\;\;k=1,\;2,\;\ldots ,\;n$$

The neighboring mean background sequence can be calculated as Equation (4).

(4) $$z^{\left(1\right)}(k)=\frac{1}{2}\left[x^{\left(1\right)}(k)+x^{\left(1\right)}(k-1)\right],\;\;\;\;k=2,\;3,\;\ldots ,\;n$$

The basic grey differential equation is then written as Equation (5) and its whitening differential equation is typically written as Equation (6).

(5) $$x^{\left(0\right)}(k)+az^{\left(1\right)}(k)=b$$

(6) $$\frac{dx^{\left(1\right)}}{dk}+ax^{\left(1\right)}=b$$

where *a* is the development coefficient and *b* is the grey action quantity. Their values depend on the construction form of the original sequence and background value, and can be determined using the least squares method:

(7) $${\left[a,b\right]}^{T}={\left[B^{T}B\right]}^{-1}B^{T}Y$$

where $Y=\left[\begin{array}{c} x^{\left(0\right)}(2)\\ x^{\left(0\right)}(3)\\ \vdots \\ x^{\left(0\right)}(n) \end{array}\right],B=\left[\begin{array}{cc} -z^{\left(1\right)}(2) & 1\\ -z^{\left(1\right)}(3) & 1\\ \vdots & \vdots \\ -z^{\left(1\right)}(n) & 1 \end{array}\right]$.

Solving differential Equation (6) yields:

(8) $$x^{\left(1\right)}(k)=\left[x^{\left(0\right)}(1)-\frac{b}{a}\right]e^{-a(k-1)}+\frac{b}{a},\;\;\;\;k=1,\;2,\;\ldots ,\;n$$

Using the regression formula for reduction calculation, the fitted value of the original data *x*^(0)^(*t*)—that is, the predicted value—is:

(9) $${\hat{x}}^{\left(0\right)}(k)=x^{\left(1\right)}(k)-x^{\left(1\right)}(k-1)=\left\{\begin{array}{cc} x^{\left(0\right)}(1), & k=1\\ (1-e^{a})\left(x^{\left(0\right)}(1)-\frac{b}{a}\right)e^{-a(k-1)}, & k=2,3,\cdots n \end{array}\right.$$

### 3.2. Markov Residual Corrected GM (1,1) Model

The Markov residual corrected GM (1,1) model combines the global trend representation of conventional GM (1,1) with residual forecasting and Markov-state identification. The method was originally proposed to improve prediction when the residual sequence contains alternating positive and negative deviations that cannot be directly handled by a non-negative grey residual model. After fitting the conventional GM (1,1), the residual sequence was calculated as Equation (10).

(10) $$\varepsilon_{1}^{\left(0\right)}(k)=x^{\left(0\right)}(k)-{\hat{x}}^{\left(0\right)}(k),\;k=2,\;3,\;\ldots ,\;n$$

Because conventional GM modeling requires a non-negative sequence, the absolute residual magnitude was defined as $\varepsilon^{\left(0\right)}(k)=\left|\varepsilon_{1}^{\left(0\right)}(k)\right|$. A second GM (1,1) model was then established for $\varepsilon^{\left(0\right)}=\left\{\varepsilon^{\left(0\right)}(2),\varepsilon^{\left(0\right)}(3),\ldots ,\varepsilon^{\left(0\right)}(n)\right\}$. Thus, the forecast residual correction value ${\hat{\varepsilon }}^{\left(0\right)}(k)$ was obtained as Equation (11).

(11) $${\hat{\varepsilon }}^{\left(0\right)}(k)=\varepsilon^{\left(1\right)}(k)-\varepsilon^{\left(1\right)}(k-1)=\left\{\begin{array}{cc} \varepsilon^{\left(0\right)}(1), & k=1\\ (1-e^{a_{\varepsilon }})\left(\varepsilon^{\left(0\right)}(1)-\frac{b_{\varepsilon }}{a_{\varepsilon }}\right)e^{-a_{\varepsilon }(k-1)}, & k=2,3,\cdots n \end{array}\right.$$

Based on Equations (9) and (11), the corrected prediction can therefore be expressed as Equation (12).

(12) $${\hat{x}}_{MR}^{\left(0\right)}(k)={\hat{x}}^{\left(0\right)}(k)+s(k){\hat{\varepsilon }}^{\left(0\right)}(k)$$

where $s(k)\in \left\{-1.+1\right\}$ denotes the predicted residual correction.

A two-state Markov chain was employed to determine *s*(*k*). The residual states were defined as Equation (13).

(13) $$S_{1}:\varepsilon_{1}^{\left(0\right)}(k)>0,S_{2}:\varepsilon_{1}^{\left(0\right)}(k)<0$$

The state-transition probability matrix was constructed as Equation (14).

(14) $$P=\left[\begin{array}{cc} p_{11} & p_{12}\\ p_{21} & p_{22} \end{array}\right]$$

where *p_ij_* represents the probability of transitioning from state *i* to state *j*, *p_ij_* ≥ 0, $\sum_{j=1}^{2}p_{ij}=1$, and *p_ij_* can be calculated as Equation (15).

(15) $$p_{ij}=\frac{s_{ij}}{s_{i}},i=1,2;j=1,2$$

where *s_ij_* represents the number of transitions from state *i* to state *j*, and *s_i_* represents the number of occurrences of state *i*.

Define $s^{\left(0\right)}=(s_{1}^{\left(0\right)},s_{2}^{\left(0\right)})$ as the initial state vector, $s_{1}^{\left(0\right)}$ as the probability of being in state 1, and $s_{2}^{\left(0\right)}$ as the probability of being in state 2. Then, using state transition Equation (16), calculate the state transition result *s*^(*t*)^ for the next time step; that is, at time *t*.

(16) $$s^{\left(t\right)}=s^{\left(0\right)}\cdot P$$

The state with higher probability in *s*^(*t*)^ is selected as the state (i.e., the sign) of the residual correction value at time *t*. If the probabilities of the two states are equal, then take the state of the residual correction value at the previous time as the state of the residual correction value at time *t*.

### 3.3. FOTP-GM (1,1) Model

The full order time power grey forecasting model, FOTP-GM (1,1), extends conventional GM (1,1) by introducing polynomial terms of the ordered sequence index. Its general form is as Equation (17).

(17) $$x^{\left(0\right)}(k)+az^{\left(1\right)}(k)=\sum_{i=1}^{h}b_{i}k^{h-i},k\in Z,k\geq 2,h\geq 1$$

where *a* is the development coefficient, *b_i_* are grey action quantities, and *h* denotes the order of the power terms. This formulation provides greater structural flexibility than conventional GM (1,1), allowing the model to represent ordered sequences that depart from a single exponential development pattern.

For parameter vector, ${\left[\begin{array}{ccccc} a & b_{1} & b_{2} & \cdots & b_{h} \end{array}\right]}^{T}$, can be determined using the least squares method:

(18) $${\left[\begin{array}{ccccc} a & b_{1} & b_{2} & \cdots & b_{h} \end{array}\right]}^{T}={\left[B^{T}B\right]}^{-1}B^{T}Y$$

where $Y=\left[\begin{array}{c} x^{\left(0\right)}(2)\\ x^{\left(0\right)}(2)\\ \vdots \\ x^{\left(0\right)}(n) \end{array}\right],\;B=\left[\begin{array}{ccccc} -z^{\left(1\right)}(2) & 2^{h-1} & 2^{h-2} & \cdots & 1\\ -z^{\left(1\right)}(3) & 3^{h-1} & 3^{h-2} & \cdots & 1\\ \vdots & \vdots & \vdots & & \vdots \\ -z^{\left(1\right)}(n) & n^{h-1} & n^{h-2} & \cdots & 1 \end{array}\right].$

To obtain the discrete response sequence, the following coefficients were introduced:

$m=\frac{1-0.5a}{1+0.5a},q=\frac{1}{1+0.5a}$

The accumulated response can then be written as Equation (19), and the original sequence is recovered through Equation (20).

(19) $${\hat{x}}^{\left(1\right)}(k)=m^{k-1}x^{\left(1\right)}(1)+q\sum_{j=1}^{h}b_{j}\sum_{i=2}^{k}m^{k-i}i^{h-j}$$

(20) $${\hat{x}}^{\left(0\right)}(k)={\hat{x}}^{\left(1\right)}(k)-{\hat{x}}^{\left(1\right)}(k-1)$$

The order *h* controls the structural flexibility of FOTP-GM(1,1). Increasing *h* generally improves in-sample fitting, but excessive model order may lead to overfitting, numerical ill-conditioning, and unstable extrapolation. Therefore, *h* was selected according to training-only forecasting performance rather than goodness of fit.

Candidate orders of *h* = 1, 2, 3, and 4 were considered. For an order-*h* FOTP model, the parameter vector contains *h* + 1 unknown coefficients, whereas a training sequence containing *n* observations provides *n* − 1 regression equations. Consequently, at least *h* + 2 observations are required for parameter estimation. In addition, because at least one observation must remain for internal forward validation, the admissible candidate set at a forecasting origin with *n* available observations was defined as $\zeta (n)=\left\{h:1\leq h\leq \min(4,n-3)\right\}$. For example, only *h* = 1 was admissible when four observations were available, whereas *h* = 1–4 could be evaluated when at least seven observations were available. This restriction prevented underdetermined or non-validated high-order models from entering the order-selection procedure.

Numerical stability was subsequently examined for each admissible candidate. A candidate was excluded when cond(*B^T^B*) ≥ 10^12^. The remaining candidates were compared using expanding-window forward validation conducted exclusively within the current training sequence. Let *h*_max_ denote the highest admissible candidate order. Internal validation started from a fitting window containing *h*_max_ + 2 observations, which ensured that all candidate orders were evaluated using identical validation targets. At each internal forecasting origin, the model was fitted using only the preceding observations and used to predict the next observation. For candidate order *h*, the internal validation error *RMSE* was calculated. The order yielding the minimum *RMSE* was selected. If two candidate orders produced essentially identical validation errors, the lower order was preferred according to the principle of parsimony. Finally, the selected FOTP model was refitted using all observations available at the forecasting origin and applied to the subsequent unseen observation(s). No test observation beyond the forecasting origin was used in either model fitting or order selection.

### 3.4. Improved Metabolic GM (1,1) Model

The improved metabolic GM (1,1) model was introduced to account for the progressive reduction in the relevance of old information as an ordered system evolves. Instead of permanently retaining the original data window, a newly predicted value is inserted into the sequence while the oldest observation is removed, thereby maintaining a constant sequence dimension.

Consider an initial sequence $X_{1}^{\left(0\right)}=\left\{x^{\left(0\right)}(1),x^{\left(0\right)}(2),\ldots ,x^{\left(0\right)}(N)\right\}$. A conventional GM (1,1) is first established from $X_{1}^{\left(0\right)}$, producing ${\hat{x}}^{\left(0\right)}(N+1)$. The updated sequence is then constructed as $X_{2}^{\left(0\right)}=\left\{x^{\left(0\right)}(2),x^{\left(0\right)}(3),\ldots ,x^{\left(0\right)}(N),{\hat{x}}^{\left(0\right)}(N+1)\right\}$, and a new GM (1,1) model is established using $X_{2}^{\left(0\right)}$ to predict ${\hat{x}}^{\left(0\right)}(N+2)$. The procedure is recursively repeated as Equation (21).

(21) $$X_{r+1}^{\left(0\right)}=\left\{X_{r}^{\left(0\right)}(2:N),{\hat{x}}^{\left(0\right)}(N+r)\right\}$$

Thus, the improved metabolic approach retains a constant modeling dimension while gradually replacing the earliest information with predicted new information.

### 3.5. Reference Benchmark Models

To provide external references for evaluating the predictive performance of the grey models, three benchmark models were additionally considered: linear regression, exponential regression, and the classical Fick-based diffusion model. The linear and exponential regression models were expressed as Equation (22) and Equation (23), respectively.

(22) $$C(s)=\beta_{0}+\beta_{1}s$$

(23) $$C(s)=\alpha e^{\beta s}$$

where *s* = *t* for temporal sequences and *s* = *x* for spatial sequences.

The classical Fick-based model was expressed as Equation (24).

(24) $$C(x,t)=C_{0}+(C_{s}-C_{0})\left[1-erf(\frac{x}{2\sqrt{D_{a}t}})\right]$$

where *C*_0_, C_s_, and *D_a_* denote the initial chloride concentration, surface chloride concentration, and apparent diffusion coefficient, respectively.

The linear model was fitted by ordinary least squares, whereas the exponential model was fitted by linear regression of ln *C* against *s*. For the Fick-based benchmark, *C*_0_ was set to zero and *Cs* and *Da* were estimated by nonlinear least squares subject to *Cs* > 0 and *Da* > 0. For temporal sequences, *x* was fixed at 10 or 14 mm, whereas for spatial sequences, *t* was fixed at 280 or 320 d. All benchmark models were refitted using only the observations available at each forecasting origin and were evaluated using exactly the same holdout, rolling one-step, and rolling two-step validation splits as the grey models. For rolling two-step forecasting, both horizons were predicted from the model established at the corresponding forecasting origin. No test observations were used for parameter estimation.

### 3.6. Unified Forecasting and Validation Strategy

To ensure a fair comparison, all grey models and reference benchmark models were evaluated using the same unified validation framework comprising holdout, rolling one-step, and rolling two-step forecasting.

#### 3.6.1. Holdout Forecasting

For the temporal sequences containing eight observations, the first six observations, 40–240 d, were used for model development, whereas the final two observations, 280 and 320 d, were retained as unseen test data. Thus, $X_{train,T}=\left\{C_{40},C_{80},\ldots ,C_{240}\right\}$ was used to predict $\left\{C_{280},C_{320}\right\}$.

For the spatial sequences containing ten observations, the concentrations measured between 6 and 34 mm were used for training, while the concentrations at 38 and 42 mm were retained for holdout testing. This procedure evaluated genuine extrapolation performance rather than the ability of a model to reproduce the data from which its parameters were estimated.

#### 3.6.2. Rolling One-Step Forecasting

Because a single holdout partition may produce results dependent on the selected training length, forward-chaining rolling validation was additionally conducted.

For temporal sequences, successive forecasting tasks were constructed as 40–160 d → 200 d, 40–200 d → 240 d, 40–240 d → 280 d, and 40–280 d → 320 d.

For spatial sequences, the procedure began with 6–18 mm → 22 mm, and the training interval was progressively extended to 6–38 mm → 42 mm.

At every forecasting origin, the model was reconstructed using only the observations located before the prediction target. For FOTP-GM, the admissible candidate orders and the optimal order were re-determined at each forecasting origin according to the training-only procedure described in Section 3.3, thereby excluding underdetermined models and preventing information leakage.

#### 3.6.3. Rolling Two-Step Forecasting

A second multi-step validation scheme was introduced for two reasons. First, deterioration or chloride-transport applications often require extrapolation beyond a single neighboring observation. Second, one-step validation cannot distinguish the improved metabolic model from conventional GM (1,1), because their first forecast from an identical data window is theoretically the same.

For example, temporal two-step forecasts included 40–160 d → {200, 240 d}, 40–200 d → {240, 280 d}, and 40–240 d → {280, 320 d}. Similarly, spatial multi-step forecasting was performed by successively predicting the next two deeper concentration values from each available shallow-to-deep training sequence.

The combination of holdout, rolling one-step, and rolling two-step validation was intended to distinguish fitting capability, local extrapolation capability and recursive multi-step forecasting capability. No measured observation located beyond a given forecasting origin was introduced into model fitting or FOTP order selection. The MATLAB 2024b code used for model fitting, FOTP order selection, validation, and performance evaluation is provided in Supplementary File S1.

### 3.7. Prediction Accuracy and Physical Applicability Criteria

#### 3.7.1. Sequence Compatibility Assessment

Before forecasting, the compatibility of each original sequence with the conventional GM(1,1) structure was examined using the level-ratio test. For an original sequence, the level ratio is defined as Equation (25).

(25) $$\lambda \left(k\right)=\frac{x^{\left(0\right)}(k-1)}{x^{0}(k)},k=2,3,\ldots ,n$$

For a sequence of length *n*, the admissible interval for conventional GM (1,1) is as Equation (26).

(26) $$e^{-2/(n+1)}<\lambda (k)<e^{2/(n+1)}$$

A sequence with a higher proportion of level ratios falling within this interval is generally considered more compatible with the quasi-exponential characteristics preferred by conventional GM(1,1). In this study, the level ratio pass rate was calculated as the percentage of $\lambda \left(k\right)$ values satisfying Equation (26) and was used as a diagnostic indicator of sequence compatibility. The level-ratio test was not used as an exclusion criterion, because the purpose of this study was to compare the forecasting performance of different grey models for sequences with distinct structural characteristics.

#### 3.7.2. Statistical Prediction Metrics

(1) Mean absolute error

The mean absolute error was calculated as Equation (27).

(27) $$MAE=\frac{1}{N}\sum_{i=1}^{N}\left|C_{i}-{\hat{C}}_{i}\right|$$

where *C_i_* and ${\hat{C}}_{i}$ are the measured and predicted chloride concentrations, respectively.

(2) Root mean square error

The root mean square error was calculated as Equation (28).

(28) $$RMSE=\sqrt{\frac{1}{N}\sum_{i=1}^{N}{\left|C_{i}-{\hat{C}}_{i}\right|}^{2}}$$

*RMSE* gives greater weight to relatively large prediction deviations and was therefore used as one of the primary model-selection criteria.

(3) Mean absolute percentage error

The mean absolute percentage error was calculated as Equation (29).

(29) $$MAPE=\frac{100}{N}\sum_{i=1}^{N}\left|\frac{C_{i}-{\hat{C}}_{i}}{C_{i}}\right|$$

*MAPE* provides an intuitive point-wise relative error measure. However, it can become highly sensitive when *C_i_* → 0. This issue is particularly relevant for spatial chloride profiles because chloride concentration may decrease by several orders of magnitude from the exposed surface toward the deep region. Consequently, *MAPE* was retained as a supplementary indicator but was not used independently to rank spatial models.

(4) Weighted absolute percentage error

To reduce the excessive influence of individual near-zero concentrations, the weighted absolute percentage error was additionally introduced:

(30) $$WAPE=100\frac{\sum_{i=1}^{N}\left|C_{i}-{\hat{C}}_{i}\right|}{\sum_{i=1}^{N}C_{i}}$$

In contrast to *MAPE*, *WAPE* normalizes the total absolute prediction error by the total measured concentration and therefore provides a more stable measure for strongly decaying chloride concentration profiles. For the spatial-direction comparison, *WAPE* and *RMSE* were regarded as primary overall-error indicators, whereas *MAPE* was mainly used to assess the sensitivity of predictions in the low-concentration tail.

(5) Normalized *RMSE*

To permit comparison between temporal and spatial sequences having different concentration ranges, *RMSE* was further normalized as Equation (31).

(31) $$NRMSE=100\frac{RMSE}{C_{\max}-C_{\min}}$$

where *C*_max_ and *C*_min_ are the maximum and minimum measured concentrations of the corresponding complete experimental sequence.

*NRMSE* enables prediction errors from different directions and concentration levels to be compared on a common dimensionless scale.

(6) For evaluation sets containing a sufficient number of observations, Equation (32) was used to assess the ability of the model to reproduce the overall variation in chloride concentration.

(32) $$R^{2}=1-\frac{\sum_{i=1}^{N}{\left(C_{i}-{\hat{C}}_{i}\right)}^{2}}{\sum_{i=1}^{N}{\left(C_{i}-{\bar{C}}_{i}\right)}^{2}}$$

Because *R*^2^ becomes highly unstable when calculated from only two holdout observations, the *R*^2^ obtained from the two-point holdout test was not used as a principal model-ranking criterion. Instead, *R*^2^ was mainly interpreted for rolling forecasts containing a larger number of independent prediction targets.

#### 3.7.3. Physical Admissibility

Chloride concentration is intrinsically non-negative:$C\left(x,t\right)\geq 0$. Therefore, any model prediction satisfying $\hat{C}\left(x,t\right)<0$ was classified as a nonphysical prediction, irrespective of its statistical error relative to the measured value.

The nonphysical prediction rate was defined as Equation (33).

(33) $$NPR=\frac{N_{\hat{C}<0}}{N_{prediction}}\times 100\%$$

To incorporate the non-negativity requirement directly into model applicability assessment, a two-stage evaluation strategy was adopted. First, physical feasibility was examined by requiring all predicted chloride concentrations to satisfy $\hat{C}\geq 0$. A model with *NPR* > 0 was therefore classified as physically inadmissible for the corresponding forecasting condition and was excluded from final model preference, irrespective of its statistical error. Second, statistical performance was compared among the physically admissible models using *MAE*, *RMSE*, *MAPE*, *WAPE*, *NRMSE*, and *R*^2^.

Negative predictions were deliberately retained rather than truncated to zero because post-processing such as $\hat{C}=\max(0,\hat{C})$ would conceal extrapolation instability and artificially improve the calculated error metrics. Thus, the non-negativity condition in this study was imposed as a feasibility criterion for model applicability rather than as a post hoc correction to the predicted values. This criterion is particularly important for deep spatial profiles, where the measured chloride concentration approaches zero and residual-correction or high-order extrapolation terms may become comparable to the baseline prediction.

All numerical analyses were performed using MATLAB R2024b (MathWorks, Natick, MA, USA).

## 4. Results

### 4.1. Direction-Dependent Characteristics of Chloride Concentration Sequences

**Figure 1:**
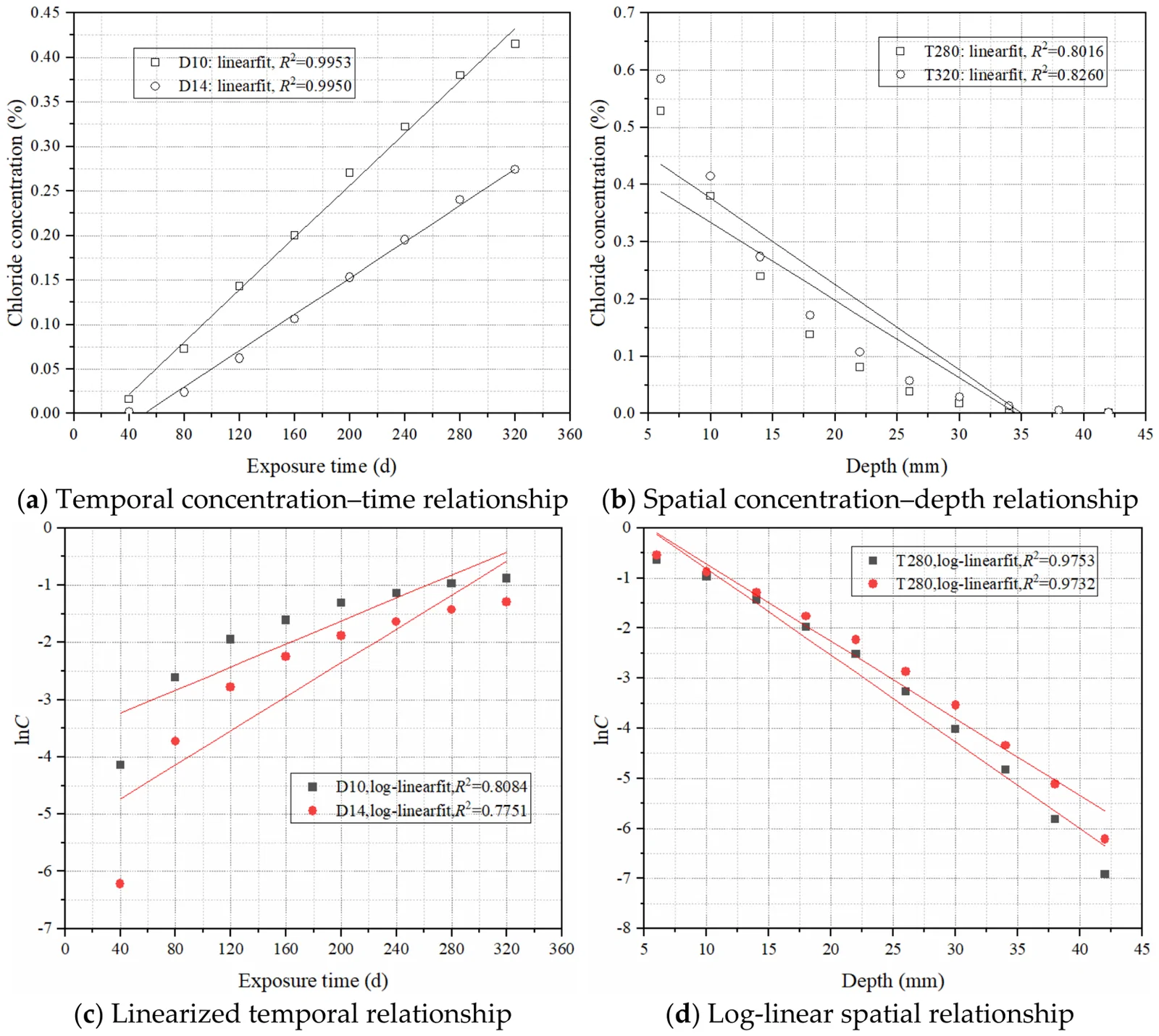
Direction-dependent characteristics of temporal and spatial chloride concentration sequences.

Before comparing the forecasting models, the intrinsic characteristics of the chloride concentration sequences in the temporal and spatial directions were examined. As shown in Figure 1, the two temporal sequences exhibited a monotonic increase in chloride concentration with exposure time, whereas the two spatial sequences showed a rapid monotonic attenuation with increasing depth.

For the temporal sequences, linear regression provided excellent representations of the measured data. The coefficients of determination were *R*^2^ = 0.9953 and *R*^2^ = 0.9950 for the chloride concentrations at depths of 10 and 14 mm, respectively. In contrast, the corresponding coefficients obtained by log-linear regression were only 0.8084 and 0.7751. These results indicate that, within the investigated exposure period of 40–320 d, chloride concentration at a fixed depth exhibited a quasi-linear increasing trend rather than a conventional exponential growth pattern.

A distinctly different sequence structure was observed in the spatial direction. The direct linear regressions of chloride concentration against depth resulted in *R*^2^ values of only 0.8016 and 0.8260 for 280 and 320 d, respectively. However, after logarithmic transformation of chloride concentration, the relationships between ln*C* and depth became highly linear, with *R*^2^ = 0.9753 at 280 d and *R*^2^ = 0.9733 at 320 d. Therefore, the spatial chloride profiles can be characterized as approximately exponential-like attenuation sequences over the investigated depth range.

The level-ratio test described in Section 3.7.1 further revealed clear differences in the compatibility of the temporal and spatial sequences with conventional GM (1,1). For the complete temporal sequences, 42.9% and 28.6% of the level ratios fell within the admissible GM (1,1) interval for the 10 and 14 mm sequences, respectively. In comparison, the pass rate was 0% for both spatial sequences. In the spatial profiles, the concentration ratios between adjacent depths increased markedly toward the interior of the concrete, indicating that the attenuation rate was substantially greater than that preferred by a conventional smooth GM (1,1) sequence. These results demonstrate that although the four sequences were extracted from the same chloride transport field *C* (*x*,*t*), their mathematical characteristics were strongly direction-dependent. The temporal sequences were dominated by quasi-linear growth, whereas the spatial sequences exhibited approximately exponential-like decay.

### 4.2. Prediction Performance in the Temporal Direction

#### 4.2.1. Holdout Forecasting at Fixed Depths

**Figure 2:**
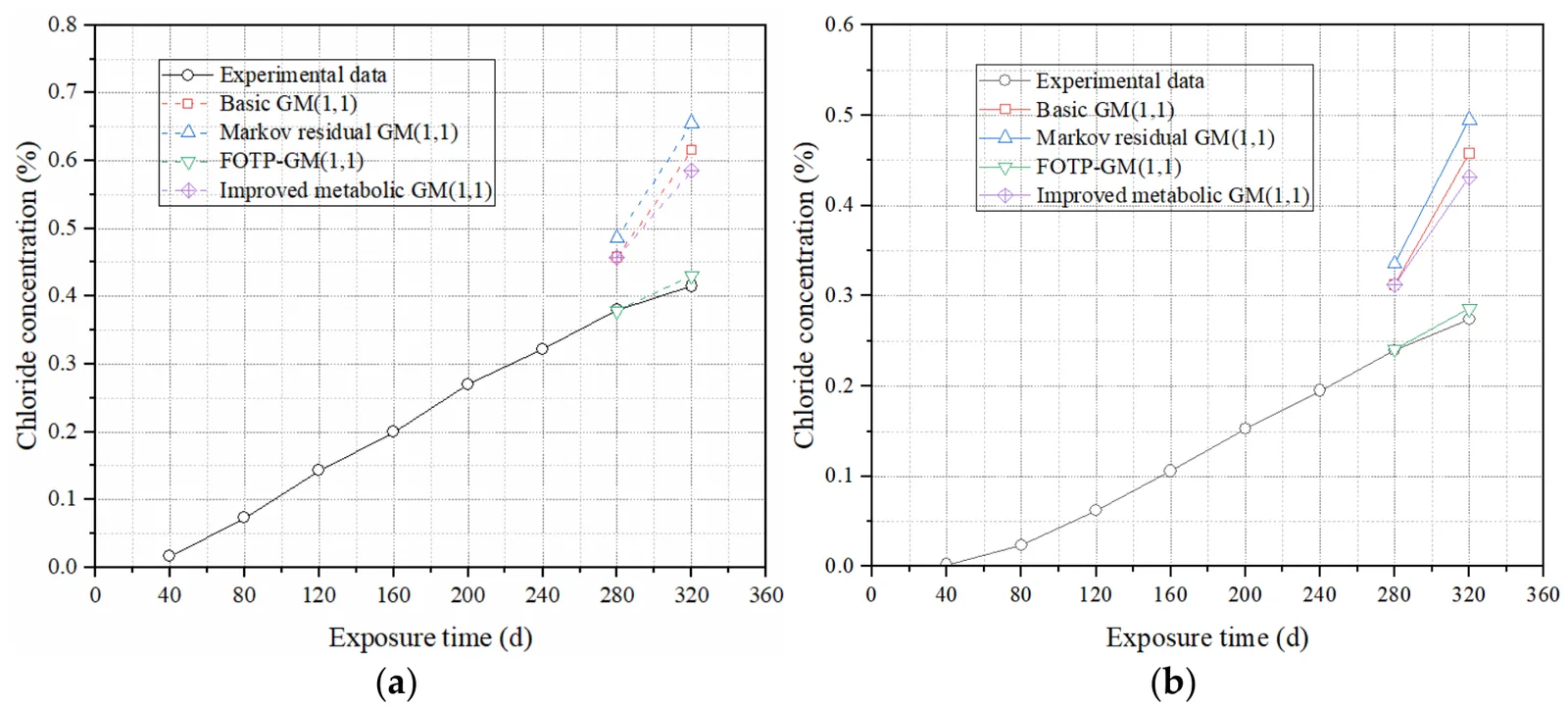
Holdout forecasting results of the four grey models for temporal chloride sequences: (**a**) 10 mm and (**b**) 14 mm.

**Figure 3:**
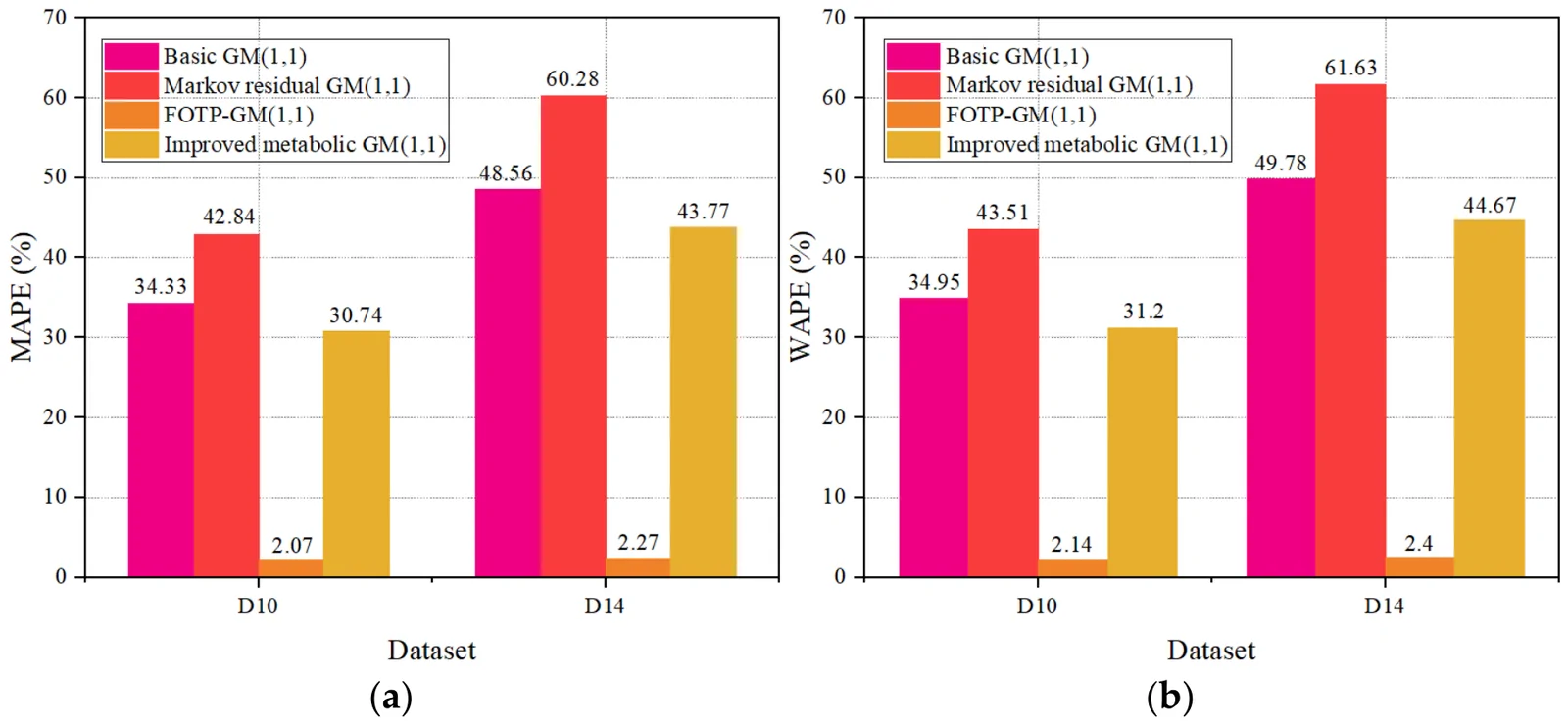
Comparison of *MAPE* and *WAPE* for holdout forecasting of temporal chloride sequences: (**a**) 10 mm and (**b**) 14 mm.

The last two exposure ages, 280 and 320 d, were first retained as unseen observations, while the measurements from 40 to 240 d were used for model development. The prediction results of the four models are shown in Figure 2. The corresponding MAPE and WAPE are shown in Figure 3.

For the 10 mm sequence, the measured chloride concentrations at 280 and 320 d were 0.380% and 0.415%, respectively. Conventional GM (1,1) predicted these values as 0.4574 and 0.6154, indicating a clear tendency to overestimate late-age chloride accumulation. The corresponding *MAPE* and *WAPE* reached 34.33% and 34.95%, respectively. Residual correction using the Markov model did not improve the prediction. The predicted concentrations increased further to 0.4858 and 0.6551, resulting in a *MAPE* of 42.84% and a *WAPE* of 43.51%. This suggests that the dominant forecasting error was not simply an oscillatory residual around an otherwise correct GM trend. The improved metabolic GM (1,1) moderately reduced the two-step prediction error. Although its first prediction was identical to that of conventional GM (1,1), the updated second prediction decreased from 0.6154 to 0.5856. Consequently, its *MAPE* and *WAPE* decreased to 30.74% and 31.20%, respectively. Nevertheless, the model still substantially overestimated the measured chloride concentration.

In contrast, FOTP-GM (1,1) showed markedly improved forecasting accuracy. The training-only order-selection procedure identified *h* = 2 as the optimal structure. The resulting concentrations at 280 and 320 d were 0.3782 and 0.4302, respectively, closely reproducing the experimental values. Its *MAPE* was only 2.07% and its *WAPE* was 2.14%, representing an approximately one-order-of-magnitude reduction in relative forecasting error compared with the other three models.

A similar pattern was observed for the 14 mm sequence. Conventional GM (1,1), Markov residual GM (1,1), and improved metabolic GM (1,1) all noticeably overpredicted the late-stage chloride concentration. Their *MAPE*s were 48.56%, 60.28%, and 43.77%, respectively. In comparison, FOTP-GM selected an optimal order of *h* = 3 and predicted concentrations of 0.2408 and 0.2855 at 280 and 320 d, compared with measured values of 0.240 and 0.274. The resulting MAPE and WAPE were only 2.27% and 2.40%, respectively.

Therefore, the excellent performance of FOTP-GM in the temporal direction was reproduced at both investigated depths, indicating that its advantage was not limited to an individual chloride concentration sequence.

#### 4.2.2. Rolling One-Step Forecasting of Temporal Profiles

**Figure 4:**
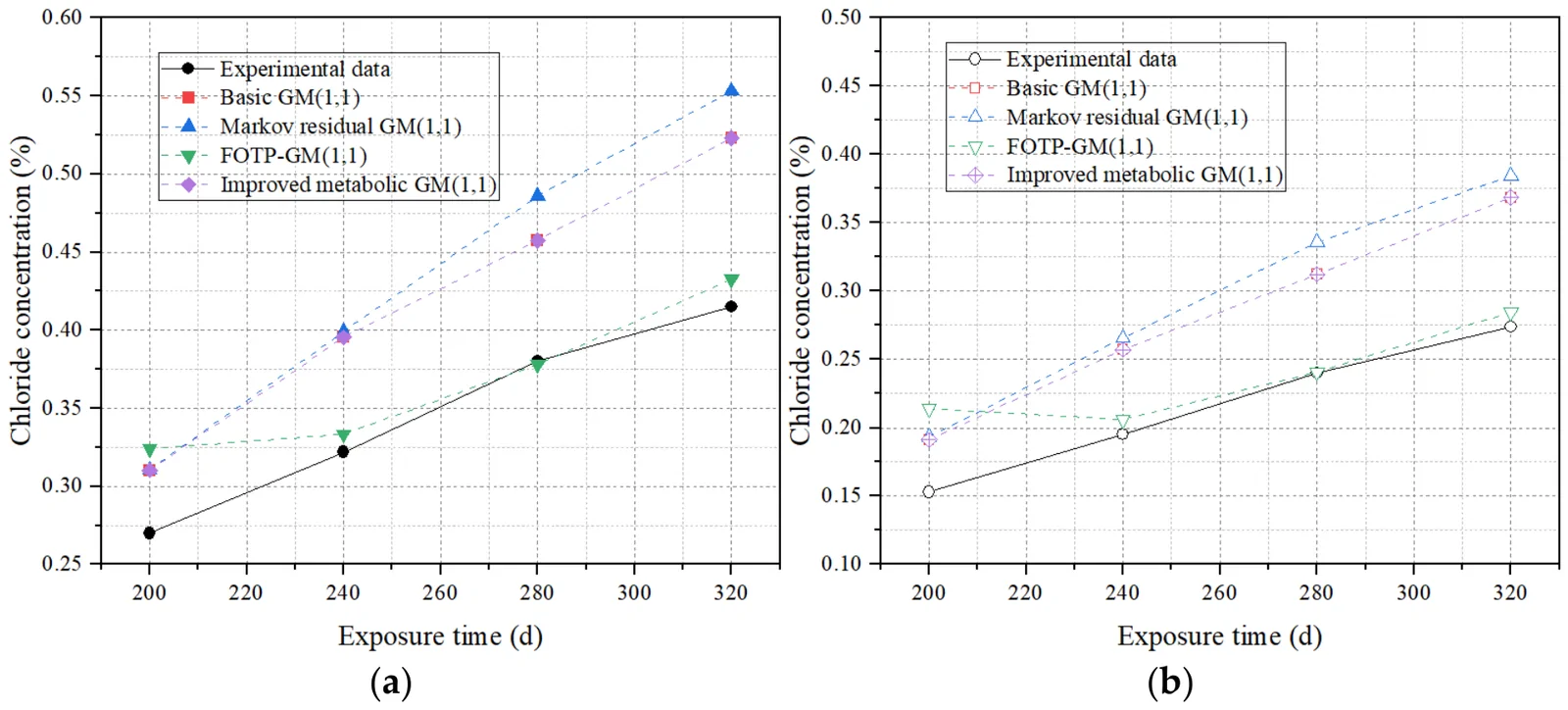
Rolling one-step forecasting results for temporal chloride sequences: (**a**) 10 mm and (**b**) 14 mm.

To determine whether the high accuracy of FOTP-GM was caused by a specific training–test partition, rolling one-step forecasting was further conducted. The prediction results are shown in Figure 4. The metrics are shown in Table 2.

For the 10 mm sequence, conventional GM (1,1) progressively overestimated the chloride concentration as the prediction origin moved forward. The corresponding rolling prediction resulted in *MAPE* = 21.0390%, *WAPE* = 21.5763% and *R*^2^ = −1.0133. Markov residual correction further increased the overall error, with a WAPE of 26.0704%. FOTP-GM maintained substantially better prediction performance throughout the rolling process. The overall rolling *MAPE* and *WAPE* were reduced to 7.0762% and 6.1273%, and *R*^2^ increased to 0.7259. The same tendency was observed at 14 mm. The *WAPE* values of conventional GM, Markov-GM, FOTP-GM, and Improved metabolic GM were 30.9772%, 36.7716%, 9.6948%, and 30.9772%, respectively. FOTP-GM also achieved the only positive and comparatively high coefficient of determination, *R*^2^ = 0.5256. Thus, FOTP-GM reduced the overall temporal forecasting error by approximately 70% relative to conventional GM (1,1). The order selected for FOTP-GM varied with the amount of temporal information available, and the values of *h* in the FOTP-GM model are shown in Table 3.

As the training sequence expanded, higher-order candidates became admissible and were selected when they reduced the internal validation error. The variation in the selected order therefore reflects both the sample-size constraint and the structural flexibility favored by training-only validation. For the holdout predictions based on the first six measurements, the optimal orders were *h* = 2 at 10 mm and *h* = 3 at 14 mm. These results are consistent with the quasi-linear but slightly curved temporal evolution observed in Section 4.1.

#### 4.2.3. Multi-Step Forecasting in the Temporal Direction

The difference between conventional and improved metabolic GM (1,1) cannot be identified from one-step forecasting because both models produce the same first prediction from an identical initial data window. Therefore, rolling two-step forecasting was performed to evaluate recursive extrapolation. The calculation results are shown in Table 4 and Table 5.

For the 10 mm sequence, the second-step *WAPE* of conventional GM (1,1) increased to 49.5019%. The Markov residual model produced an even greater *WAPE* of 53.2737%. The metabolic updating strategy reduced the second-step *WAPE* to 40.2964%, confirming that replacing old information with newly predicted values partially alleviated the accumulation of extrapolation error. Nevertheless, FOTP-GM retained the highest accuracy, with a second-step *WAPE* of only 19.6915%.

Similar results were obtained at 14 mm. The second-step *WAPE* values were 74.0768%, 81.008%, 35.4792%, and 58.7576% for conventional GM, Markov-GM, FOTP-GM, and metabolic GM, respectively. Therefore, although the predictive advantage of FOTP-GM decreased as the forecasting horizon increased, it remained clearly superior to the other models for temporal chloride concentration extrapolation. This result also confirms that the improvement introduced by the metabolic mechanism becomes visible primarily in multi-step forecasting rather than in single-step prediction.

### 4.3. Prediction Performance in the Spatial Direction

#### 4.3.1. Holdout Prediction of Deep Chloride Concentrations

**Figure 5:**
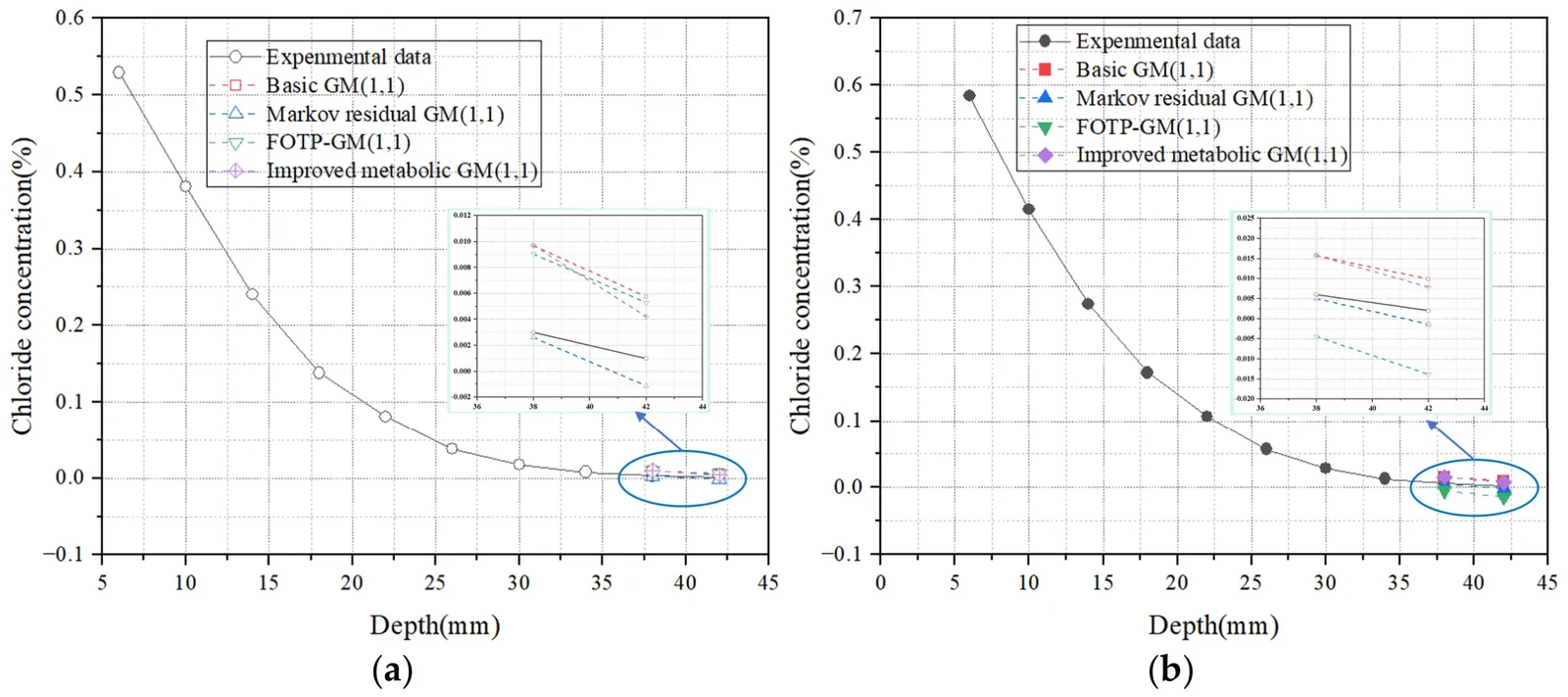
Holdout forecasting results of the four grey models for spatial chloride profiles: (**a**) 280 d and (**b**) 320 d.

A substantially different model response was obtained for the spatial sequences. The concentrations measured from 6 to 34 mm were used for model establishment, while those at 38 and 42 mm were retained for independent validation. The prediction results of the four models are shown in Figure 5. The corresponding metrics are shown in Table 6.

At 280 d, the measured concentrations at 38 and 42 mm were only 0.003 and 0.001. Conventional GM (1,1) predicted 0.0097 and 0.0057, respectively. Although the absolute deviations were small, the corresponding *MAPE* reached 348.5166% because the measured concentrations approached zero. FOTP-GM selected *h* = 1 and predicted 0.0090 and 0.0053. Its *MAPE* remained high at 314.8732%, showing that the introduction of additional power terms was unnecessary for this spatial sequence. The improved metabolic model slightly reduced the second predicted concentration to 0.0042, but its *MAPE* was still 272.7015%. Markov residual correction substantially reduced the absolute error at 38 mm, giving a prediction of 0.0026. However, its prediction at 42 mm became −0.00113, which is physically impossible for chloride concentration. Therefore, despite its lower *MAE* and *RMSE*, the Markov model could not be considered physically reliable in the low-concentration region.

The instability became more pronounced for the 320 d spatial profile. FOTP GM selected *h* = 2, but its two holdout predictions were −0.0044 and −0.0139, resulting in a nonphysical prediction rate of 100%. Markov GM also generated a negative prediction at 42 mm. In contrast, conventional and metabolic GM remained non-negative, although both considerably overpredicted the deep chloride concentrations.

These findings show that small overall absolute errors in a spatial concentration profile do not necessarily imply reliable extrapolation of the low-concentration tail.

#### 4.3.2. Rolling One-Step Forecasting of Spatial Profiles

**Figure 6:**
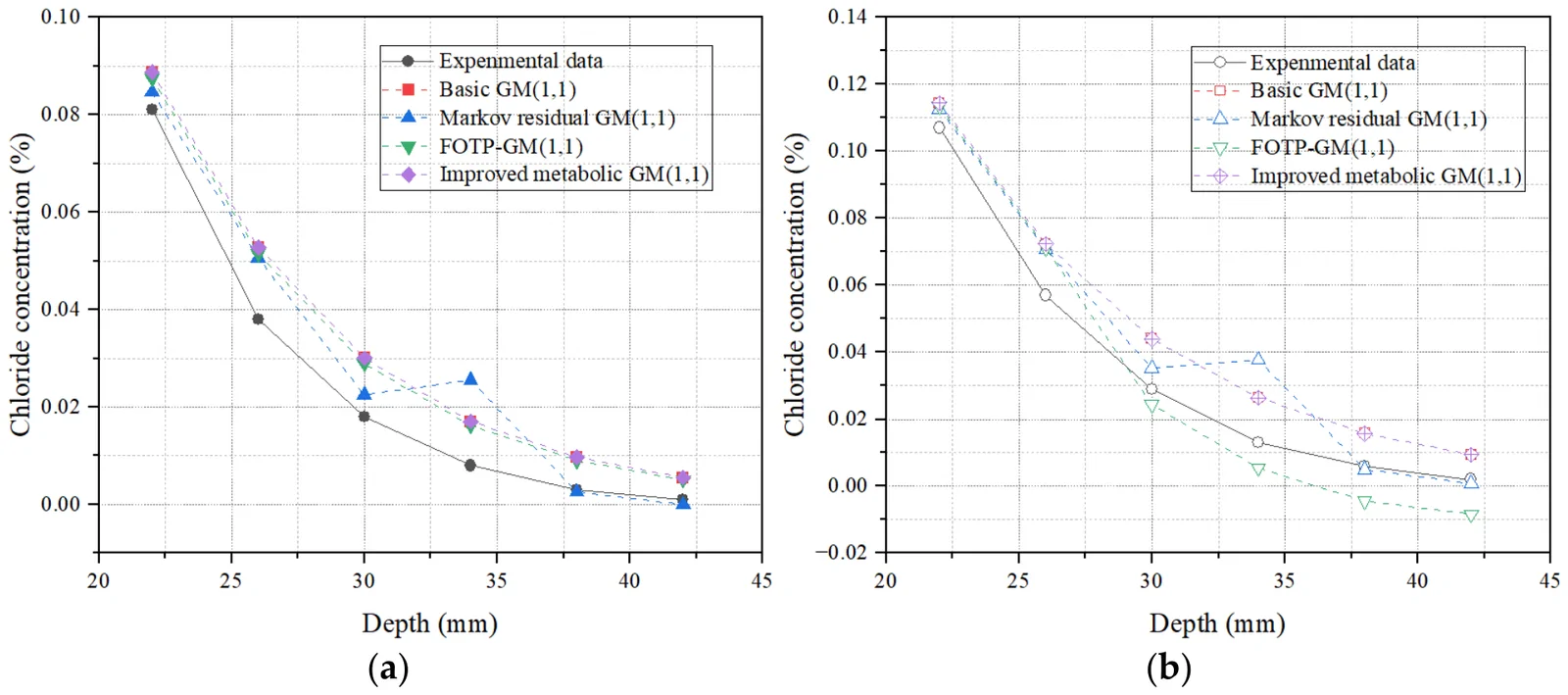
Rolling one-step forecasting results for spatial chloride profiles: (**a**) 280 d and (**b**) 320 d.

Rolling one-step validation provided a more comprehensive assessment of spatial forecasting performance. The prediction results of the four models are shown in Figure 6. The corresponding metrics are shown in Table 7.

For the 280 d sequence, conventional GM (1,1) achieved *RMSE* = 0.0097, *WAPE* = 36.7653, and *R*^2^ = 0.8789. FOTP-GM slightly improved the overall trend prediction, with *RMSE* = 0.0088, WAPE = 32.9497, and *R*^2^ = 0.9019. Importantly, the optimal FOTP order remained *h* = 1 at every forecasting origin. Thus, increasing structural complexity was not required to describe the 280 d depth profile. Markov GM produced the lowest *WAPE*, 26.5531% and a comparable *R*^2^ = 0.8940. However, one of the six rolling predictions was negative, corresponding to a nonphysical prediction rate of 16.67%. At 320 d, FOTP-GM provided the highest overall coefficient of determination, *R*^2^ = 0.9340, and the lowest *RMSE* = 0.0095. Its WAPE of 25.0379% was close to the 24.5384% obtained by Markov GM and lower than the 31.9010% of conventional GM. Nevertheless, FOTP-GM produced negative chloride concentrations at 38 and 42 mm, giving a nonphysical prediction rate of 33.33%. Therefore, the spatial results produced two different model rankings depending on the evaluation criterion. Markov-GM and FOTP-GM generally provided smaller statistical errors, whereas conventional and metabolic GM were more robust in maintaining the non-negative physical constraint.

The optimal FOTP orders in the spatial direction were generally lower than those obtained for temporal forecasting. For the 280 d sequence, *h* = 1 was selected throughout all rolling prediction stages. For the 320 d profile, *h* = 1 was selected for the shallower forecasting origins, after which the optimum increased to *h* = 2. For the holdout analysis, *h* = 1 and *h* = 2 were selected for 280 and 320 d, respectively. The FOTP condition-number analysis further showed a rapid deterioration of numerical conditioning with increasing model order. For example, for the 280 d holdout training sequence, $cond\left(B^{T}B\right)$ increased from approximately 1.09 × 10^2^ at *h* = 1 to 4.52 × 10^3^, 2.60 × 10^6^, and 1.15 × 10^10^ for *h* = 2, 3, 4, respectively. At the same time, the training-only validation RMSE increased from 0.01 at *h* = 1 to 0.09 at *h* = 4.

Therefore, the spatial results provide clear evidence that increasing the order of FOTP-GM does not necessarily improve prediction and may substantially reduce numerical and extrapolation stability.

#### 4.3.3. Multi-Step Forecasting in the Spatial Direction

The rolling two-step forecasting results are presented in Table 8 and Table 9, revealing a distinct prediction pattern compared with that observed in the temporal direction. For the 280 d spatial sequence, the second-step *WAPE* of conventional GM increased to 78.0456%. The corresponding values for Markov-GM and FOTP-GM were 68.8463% and 71.5919%, respectively. In comparison, the improved metabolic GM reduced the second-step *WAPE* to 60.0972%. A similar result was obtained for the 320 d sequence. The second-step *WAPE* decreased from 64.5595% for conventional GM to 53.6289% for improved metabolic GM. Markov-GM produced a *WAPE* of approximately 60.9979%, while FOTP-GM increased to approximately 71.4762% and produced negative concentrations for 40% of the second-step forecasts.

Thus, unlike the temporal direction where FOTP-GM remained dominant under multi-step forecasting, the metabolic updating mechanism became more advantageous for spatial multi-step extrapolation. This result is particularly noteworthy because no improvement of metabolic GM could be observed from rolling one-step validation. The advantage emerged only after recursive updating of the data window.

### 4.4. Comparison with Reference Benchmark Models

To assess whether the increased complexity of the modified grey models provided meaningful predictive benefits, the three reference benchmark models were evaluated using the same validation splits. Table 10 compares their *WAPE* values with those of the grey model yielding the lowest *WAPE* under each forecasting condition. The second-step results were used to represent rolling two-step forecasting because they provide the more demanding extrapolation test. Complete pointwise prediction outputs for all models under the holdout, rolling one-step, and rolling two-step validation schemes are provided in Supplementary Table S1.

For the temporal sequences, FOTP-GM retained the lowest holdout *WAPE* among all compared models, with values of 2.1393% and 2.4023% at 10 and 14 mm, respectively. However, its advantage did not persist under rolling validation. At 10 mm, linear regression reduced the rolling one-step and second-step *WAPEs* to 3.9455% and 4.8160%, compared with 6.1273% and 19.6915% for FOTP-GM. At 14 mm, the Fick-based model achieved the lowest corresponding WAPEs of 2.2169% and 4.4789%. In contrast, exponential regression performed poorly for the temporal sequences, consistent with their quasi-linear rather than exponential growth characteristics.

A clearer distinction was observed for spatial forecasting. The Fick-based model consistently achieved the lowest WAPE under all validation schemes, ranging from 4.5041% to 17.7866%. Exponential regression provided intermediate performance, whereas linear extrapolation was highly unstable and generated negative concentrations in the deep region, with NPR values of 83.33–100% depending on the validation condition. These results are consistent with the approximately exponential-like attenuation of the spatial chloride profiles and the diffusion-based structure of the Fick model.

Overall, the benchmark comparison demonstrates that increased model complexity does not necessarily lead to improved extrapolation. The benefit of the modified grey models depends on the forecasting condition and the compatibility between model structure and sequence characteristics.

## 5. Discussion

### 5.1. Prediction Behavior and Physical Reliability in the Low-Concentration Tail

The spatial forecasting results reveal that model evaluation becomes particularly challenging in the deep region where chloride concentration approaches zero. In this region, small absolute deviations can be translated into extremely large percentage errors. For example, when the measured chloride concentration is on the order of 10^−3^, a prediction deviation of only several thousandths may produce a *MAPE* exceeding 100%. Therefore, the very large *MAPE* values observed in the deep spatial profiles do not necessarily indicate equally large errors in the overall concentration-depth distribution.

This explains why some models simultaneously exhibited relatively high *R*^2^ values and large percentage errors. The former mainly reflects their ability to capture the dominant attenuation trend over the entire profile, whereas the latter is strongly controlled by the near-zero concentrations in the deep region. For spatial chloride profiles spanning more than two orders of magnitude, *MAPE* alone is therefore insufficient for model ranking. *RMSE* and *WAPE* provide a more stable measure of the overall prediction error, while *MAPE* is more appropriately regarded as an indicator of local sensitivity in the low-concentration tail.

More importantly, the deep region exposed a distinction between statistical accuracy and physical reliability. Both Markov GM and FOTP-GM generated negative concentrations under some spatial forecasting conditions. Although these predictions may still yield relatively small *RMSE* or *WAPE* because the measured concentrations are themselves close to zero, they violate the fundamental physical constraint *C*(*x*,*t*) ≥ 0. Such predictions should therefore not be regarded as acceptable solely on the basis of statistical error metrics. Simply truncating negative predictions to zero would also be inappropriate, because this would conceal model instability and artificially improve the reported accuracy.

The results therefore demonstrate the necessity of applying physical admissibility as a prerequisite for model selection rather than as an auxiliary diagnostic. Under the two-stage evaluation adopted in this study, any model producing *NPR* > 0 is regarded as physically infeasible for the corresponding forecasting condition, even if it yields relatively low RMSE or WAPE. Statistical accuracy is subsequently compared only among physically admissible candidates. This treatment prevents models with mathematically small errors but physically impossible predictions from being favored in the deep low-concentration region. In particular, the deep low-concentration region should be treated as a critical test of extrapolation robustness rather than as an ordinary continuation of the overall profile. A model that reproduces the global attenuation trend but becomes unstable as *C* → 0 may still be suitable for profile reconstruction within the measured range, but should be used cautiously for deep-region extrapolation.

### 5.2. Direction-Dependent Applicability of the Grey Forecasting Models

A key finding of this study is that the applicability of grey forecasting models depends strongly on the direction in which the chloride concentration field is represented. Although the temporal and spatial sequences were extracted from the same physical transport process, their mathematical characteristics were fundamentally different. The temporal sequences at fixed depths showed quasi-linear growth, whereas the spatial sequences at fixed exposure times exhibited approximately exponential-like attenuation.

This distinction directly affected the relative performance of the four grey models. Conventional GM (1,1) is governed by an exponential-type response and therefore showed a structural mismatch with the temporal chloride sequences. The systematic overestimation observed at later exposure ages indicates that the main error did not originate from random fluctuations, but from the inability of the basic GM structure to reproduce the actual temporal evolution pattern.

FOTP-GM improved this limitation by introducing ordered-index power terms into the grey action function. The higher-order structures selected for the temporal sequences provided sufficient flexibility to describe quasi-linear and weakly nonlinear concentration growth, resulting in a substantial reduction in forecasting error. In contrast, the spatial profiles generally required only *h* = 1 or *h* = 2. Increasing the order beyond this level did not provide a consistent improvement and was accompanied by rapidly increasing condition numbers and reduced extrapolation stability.

These results demonstrate that the optimum grey-model structure is not universal even when the predicted physical variable remains unchanged. This point has important implications for the transfer of grey models between different durability problems. A model that performs well for a temporal degradation sequence should not automatically be assumed to be suitable for a spatial transport sequence, even when both are monotonic and derived from the same material system. Before model selection, the structural characteristics of the ordered sequence should therefore be examined, including its growth or attenuation pattern, level-ratio behavior, and required model complexity.

It should be noted that the present comparison was based on the 28-d curing condition and a limited number of representative temporal and spatial sequences. Therefore, the observed direction-dependent behavior should be interpreted within the investigated material, curing, and exposure conditions. Broader generalization requires further validation using additional datasets and curing conditions.

### 5.3. Influence of Model Modification Strategy and Forecasting Horizon

The results further show that the three improved grey models do not represent interchangeable alternatives to conventional GM (1,1), because each modifies a different aspect of the original model.

Markov residual corrected GM (1,1) preserves the basic GM response and attempts to improve prediction by correcting the magnitude and sign of the residual. This strategy is most effective when the baseline model has already captured the dominant trend and the remaining errors mainly represent local fluctuations. This condition was partially satisfied for the spatial sequences, where Markov correction generally reduced the overall *WAPE*. In the temporal direction, however, the baseline GM exhibited a systematic trend bias. Under such conditions, the residual contains information associated with model-form error rather than merely random deviation, and residual correction cannot fundamentally eliminate the structural mismatch. This explains why Markov-GM did not improve temporal prediction and, in some cases, further increased the forecasting error.

FOTP-GM modifies the model structure more fundamentally. By introducing power terms of the ordered index, it provides additional degrees of freedom to represent sequences that deviate from the conventional exponential response. Its strong advantage in temporal forecasting therefore reflects a correction of model form rather than simply a reduction in residual error. However, the spatial results also demonstrate that greater flexibility is not inherently beneficial. Once the order exceeds that required by the sequence structure, numerical conditioning deteriorates and extrapolation may become unstable. Therefore, *h* should be treated as a data-dependent complexity parameter and selected according to forecasting performance rather than fitting accuracy.

The improved metabolic GM exhibits yet another mechanism. Its first prediction is identical to that of conventional GM (1,1), because both models are constructed from the same initial data window. Consequently, rolling one-step forecasting cannot reveal any advantage of the metabolic strategy. The difference appears only from the second step onward, after the oldest value has been removed and the newly predicted value has been incorporated. The reduction in several two-step forecasting errors confirms that metabolic updating can partially mitigate error accumulation during recursive prediction.

These observations show that model performance depends not only on sequence characteristics, but also on the specific mechanism of model improvement and the forecasting horizon. Accordingly, an appropriate grey-model selection strategy should consider sequence structure, model modification mechanism, forecasting horizon and physical reliability, rather than ranking models according to a single error metric or a single validation scheme. For chloride transport prediction, this framework provides a more rational basis for distinguishing when structural modification, residual correction, or dynamic information updating is most appropriate.

The benchmark comparison further demonstrates that greater model complexity does not necessarily provide superior predictive performance. FOTP-GM showed a clear advantage among the grey models and retained the best performance for temporal holdout forecasting, whereas simple linear regression and the Fick-based model were more competitive under rolling temporal forecasting. For spatial extrapolation, the Fick-based model consistently outperformed both the grey models and the simple regression benchmarks, reflecting the compatibility between its diffusion-based formulation and the concentration–depth profiles. These findings indicate that the modified grey models should be regarded as complementary small-sample forecasting approaches rather than universally superior alternatives to simpler statistical or physics-based models.

## 6. Conclusions

(1) Under the investigated 28-d curing and exposure conditions, the grey forecasting models exhibited clear direction-dependent applicability. Temporal chloride concentration sequences at fixed depths showed quasi-linear growth, whereas spatial concentration profiles at fixed exposure times exhibited approximately exponential-like attenuation. These results indicate that model suitability is strongly associated with the mathematical structure of the ordered sequence.

(2) Among the investigated grey models, FOTP-GM (1,1) showed the most consistent advantage for temporal chloride forecasting. By introducing ordered-index power terms, it effectively compensated for the structural mismatch between conventional GM (1,1) and the quasi-linear temporal evolution of chloride concentration, resulting in substantially lower holdout and rolling prediction errors than conventional GM, Markov-GM, and metabolic GM.

(3) Spatial forecasting requires a joint assessment of statistical accuracy and physical reliability. Although Markov-GM and FOTP-GM could reduce overall prediction errors, both exhibited instability in the deep low-concentration region, including negative chloride concentrations. Hence, *RMSE*, *WAPE*, numerical stability, and the non-negative physical constraint should be considered together, rather than relying on *MAPE* or *R*^2^ alone.

(4) Different model structures are advantageous under different forecasting conditions. The benchmark comparison showed that increased model complexity does not universally improve forecasting performance. Model selection should therefore consider sequence characteristics, forecasting horizon, physical admissibility, and the compatibility between model structure and the target sequence.

## Figures and Tables

**Table 1:** Experimental chloride concentration sequences used for grey-model evaluation.

Temporal Directional Data				Spatial Direction Data			
*k_T_*	Exposure Time (d)	Chloride Concentration (%)		*k_S_*	Depth (mm)	Chloride Concentration (%)	
		10 mm	14 mm			280 d	320 d
1	40	0.016	0.002	1	6	0.529	0.584
2	80	0.073	0.024	2	10	0.380	0.415
3	120	0.143	0.062	3	14	0.240	0.274
4	160	0.200	0.106	4	18	0.138	0.172
5	200	0.27	0.153	5	22	0.081	0.107
6	240	0.322	0.195	6	26	0.038	0.057
7	280	0.38	0.24	7	30	0.018	0.029
8	320	0.415	0.274	8	34	0.008	0.013
				9	38	0.003	0.006
				10	42	0.001	0.002

**Table 2:** Performance metrics of rolling one-step forecasting for temporal chloride sequences.

Depth (mm)	Model	*MAE* (%)	*RMSE* (%)	*MAPE* (%)	*WAPE* (%)	*NRMSE* (%)	*R* ^2^	*NPR* (%)
10	Basic GM	0.0748	0.0786	21.0390	21.5763	19.6932	1.0133	0.00
	Markov GM	0.0904	0.0973	25.0260	26.0704	24.3802	2.0857	0.00
	FOTP GM	0.0212	0.0290	7.0765	6.1273	7.2665	0.7259	0.00
	Metabolic GM	0.0748	0.0786	21.0390	21.5763	19.6932	1.0133	0.00
14	Basic GM	0.0668	0.0697	30.3519	30.9772	25.6418	1.3306	0.00
	Markov GM	0.0792	0.0836	35.6800	36.7716	30.7268	2.3465	0.00
	FOTP GM	0.0209	0.0315	12.4466	9.6948	11.5685	0.5256	0.00
	Metabolic GM	0.0668	0.0697	30.3519	30.9772	25.6418	1.3306	0.00

**Table 3:** Admissible and selected FOTP orders for rolling one-step temporal forecasting.

**Time (d)**	200	240	280	320
**Training observations, *n***	4	5	6	7
**Admissible orders**	1	1–2	1–3	1–4
**Depth = 10 mm**	1	2	2	4
**Depth = 14 mm**	1	2	3	3

**Table 4:** Rolling two-step predictions for temporal chloride concentration sequences.

Depth (mm)	Horizon	Time (d)	Actual (%)	Basic GM (%)	Markov GM (%)	FOTP GM (%)	Metabolic GM (%)	*h*
10	1	200	0.270	0.310	0.311	0.324	0.310	1
	2	240	0.322	0.483	0.483	0.508	0.444	1
	1	240	0.322	0.395	0.399	0.334	0.395	2
	2	280	0.38	0.571	0.574	0.398	0.537	2
	1	280	0.38	0.457	0.486	0.378	0.457	2
	2	320	0.415	0.615	0.655	0.430	0.586	2
14	1	200	0.153	0.191	0.193	0.214	0.191	1
	2	240	0.195	0.359	0.360	0.410	0.311	1
	1	240	0.195	0.257	0.266	0.206	0.257	2
	2	280	0.240	0.418	0.428	0.265	0.383	2
	1	280	0.240	0.312	0.336	0.241	0.312	3
	2	320	0.274	0.458	0.495	0.286	0.431	3

**Table 5:** Performance metrics of rolling two-step forecasting for temporal chloride sequences.

Depth (mm)	Horizon	Model	*MAE* (%)	*RMSE* (%)	*MAPE* (%)	*WAPE* (%)	*NRMSE* (%)	*R* ^2^	*NPR* (%)
10	1	Basic GM	0.0637	0.0659	19.3708	19.6689	16.5078	−1.1491	0.00
	1	Markov GM	0.0745	0.0791	22.2775	22.9959	19.8327	−2.1020	0.00
	1	FOTP GM	0.0224	0.0319	8.0163	6.9259	7.9918	0.4963	0.00
	1	Metabolic GM	0.0637	0.0659	19.3708	19.6689	16.5078	−1.1491	0.00
	2	Basic GM	0.1843	0.1851	49.5704	49.5019	46.3817	−22.2841	0.00
	2	Markov GM	0.1984	0.2010	52.9735	53.2737	50.3671	−26.4574	0.00
	2	FOTP GM	0.0733	0.1085	22.1284	19.6915	27.2016	−7.0086	0.00
	2	Metabolic GM	0.1500	0.1514	40.1548	40.2964	37.9466	−14.5852	0.00
14	1	Basic GM	0.0575	0.0593	28.9799	29.3505	21.7901	−1.7835	0.00
	1	Markov GM	0.0689	0.0725	34.1473	35.1377	26.6476	−3.1629	0.00
	1	FOTP GM	0.0243	0.0358	15.2951	12.3946	13.1640	−0.0159	0.00
	1	Metabolic GM	0.0575	0.0593	28.9799	29.3505	21.7901	−1.7835	0.00
	2	Basic GM	0.1751	0.1753	75.0224	74.0768	64.4382	−28.3444	0.00
		Markov GM	0.1914	0.1928	81.2408	81.0080	70.8961	−34.5207	0.00
		FOTP GM	0.0838	0.1253	41.6415	35.4792	46.0777	−14.0044	0.00
		Metabolic GM	0.1389	0.1399	58.8616	58.7576	51.4445	−17.7032	0.00

**Table 6:** Performance metrics of holdout forecasting for spatial chloride profiles.

Exposure Time (d)	Model	*MAE* (%)	*RMSE* (%)	*MAPE* (%)	*WAPE* (%)	*NRMSE* (%)	*NPR* (%)
280	Basic GM	0.0057	0.0058	348.5166	285.8660	1.0986	0.00
	Markov GM	0.0013	0.0015	112.6843	62.7310	0.2893	50.00
	FOTP-GM	0.0052	0.0052	314.8732	258.1247	0.9918	0.00
	Metabolic GM	0.0050	0.0053	272.7015	247.9585	0.9952	0.00
320	Basic GM	0.0088	0.0089	278.3884	220.6453	1.5252	0.00
	Markov GM	0.0022	0.0025	92.5195	54.4944	0.4268	50.00
	FOTP-GM	0.0131	0.0134	482.8143	327.9515	2.3024	100.00
	Metabolic GM	0.0078	0.0081	228.4065	195.6544	1.3857	0.00

**Table 7:** Performance metrics of rolling one-step forecasting for spatial chloride profiles.

Exposure Time (d)	Model	*MAE* (%)	*RMSE* (%)	*MAPE* (%)	*WAPE* (%)	*NRMSE* (%)	*R* ^2^	*NPR* (%)
280	Basic GM	0.0091	0.0097	150.4710	36.7653	1.8455	0.8789	0.00
	Markov GM	0.0066	0.0091	65.8800	26.5531	1.7266	0.8940	16.67
	FOTP-GM	0.0082	0.0088	135.5239	32.9497	1.6604	0.9019	0.00
	Metabolic GM	0.0091	0.0097	150.4710	36.7653	1.8455	0.8789	0.00
320	Basic GM	0.0114	0.0119	120.5431	31.9010	2.0370	0.8961	0.00
	Markov GM	0.0088	0.0121	52.7982	24.5384	2.0771	0.8920	0.00
	FOTP-GM	0.0089	0.0095	132.3591	25.0379	1.6250	0.9339	33.33
	Metabolic GM	0.0114	0.0119	120.5431	31.9010	2.0370	0.8961	0.00

**Table 8:** Rolling two-step predictions for spatial chloride concentration profiles.

Exposure Time (d)	Horizon	Depth (mm)	Actual (%)	BasicGM (%)	Markov-GM (%)	FOTP-GM (%)	Metabolic GM (%)
280	1	22	0.081	0.089	0.085	0.087	0.089
	2	26	0.038	0.055	0.052	0.054	0.052
	1	26	0.038	0.053	0.051	0.051	0.053
	2	30	0.018	0.032	0.031	0.031	0.030
	1	30	0.018	0.030	0.023	0.029	0.030
	2	34	0.008	0.018	0.010	0.017	0.015
	1	34	0.008	0.017	0.026	0.016	0.017
	2	38	0.003	0.010	0.019	0.010	0.008
	1	38	0.003	0.010	0.003	0.009	0.010
	2	42	0.001	0.006	−0.001	0.005	0.004
320	1	22	0.107	0.114	0.112	0.114	0.114
	2	26	0.057	0.075	0.074	0.074	0.072
	1	26	0.057	0.072	0.071	0.071	0.072
	2	30	0.029	0.047	0.046	0.046	0.045
	1	30	0.029	0.044	0.035	0.024	0.044
	2	34	0.013	0.028	0.018	0.001	0.025
	1	34	0.013	0.026	0.038	0.005	0.026
	2	38	0.006	0.017	0.029	−0.010	0.014
	1	38	0.006	0.016	0.005	−0.004	0.016
	2	42	0.002	0.010	−0.001	−0.014	0.008

**Table 9:** Performance metrics of rolling two-step forecasting for spatial chloride profiles.

Exposure Time (d)	Horizon	Model	*MAE* (%)	*RMSE* (%)	*MAPE* (%)	*WAPE* (%)	*NRMSE* (%)	*R* ^2^	*NPR* (%)
280	1	Basic GM	0.0101	0.0105	90.3913	33.9672	1.9852	0.8634	0.00
	1	Markov GM	0.0077	0.0100	58.8590	26.0501	1.8894	0.8762	0.00
	1	FOTP-GM	0.0090	0.0094	81.4727	30.4306	1.7861	0.8894	0.00
	1	Metabolic GM	0.0101	0.0105	90.3913	33.9672	1.9852	0.8634	0.00
	2	Basic GM	0.0106	0.0115	192.5694	78.0465	2.1781	0.2790	0.00
	2	Markov GM	0.0094	0.0111	175.9267	68.8463	2.1091	0.3240	20.00
	2	FOTP-GM	0.0097	0.0106	174.9488	71.5919	2.0034	0.3900	0.00
	2	Metabolic GM	0.0082	0.0091	136.4735	60.0972	1.7164	0.5523	0.00
320	1	Basic GM	0.0122	0.0126	70.2843	28.6941	2.1570	0.8833	0.00
	1	Markov GM	0.0103	0.0132	51.6032	24.2154	2.2735	0.8704	0.00
	1	FOTP-GM	0.0087	0.0093	55.5732	20.4035	1.5934	0.9363	20.00
	1	Metabolic GM	0.0122	0.0126	70.2843	28.6941	2.1570	0.8833	0.00
	2	Basic GM	0.0138	0.0144	156.0290	64.5595	2.4680	0.4866	0.00
	2	Markov GM	0.0131	0.0151	137.0161	60.9979	2.5991	0.4306	20.00
	2	FOTP-GM	0.0153	0.0154	245.7738	71.4762	2.6473	0.4092	40.00
	2	Metabolic GM	0.0115	0.0121	121.1090	53.6289	2.0820	0.6346	0.00

**Table 10:** *WAPE* comparison between the grey model with the lowest statistical WAPE and reference benchmark models under identical validation schemes.

Dataset	Validation	Grey Model with Lowest *WAPE*	Grey *WAPE* (%)	Linear *WAPE* (%)	Exponential *WAPE* (%)	Fick *WAPE* (%)
10 mm	Holdout	FOTP-GM	2.1393	5.5550	182.1530	4.5362
10 mm	Rolling one-step	FOTP-GM	6.1273	3.9455	120.4076	3.9826
10 mm	Rolling two-step (H2)	FOTP-GM	19.6915	4.8160	275.3623	7.1028
14 mm	Holdout	FOTP-GM	2.4023	2.7719	434.0613	3.2604
14 mm	Rolling one-step	FOTP-GM	9.6948	4.5288	258.1459	2.2169
14 mm	Rolling two-step (H2)	FOTP-GM	35.4792	6.3725	753.1866	4.4789
280 d	Holdout	Markov-GM *	62.7310	9364.2857 *	120.1759	17.7866
280 d	Rolling one-step	Markov-GM *	26.5531	519.0913 *	29.5704	4.6744
280 d	Rolling two-step (H2)	Metabolic GM	60.0972	1529.0056 *	73.7890	10.0128
320 d	Holdout	Markov-GM *	54.4944	4826.0417 *	99.2197	6.2167
320 d	Rolling one-step	Markov-GM	24.5384	365.7035 *	24.4759	4.9033
320 d	Rolling two-step (H2)	Metabolic GM	53.6289	979.6417 *	58.5713	4.5041

Note: The best-performing grey model was identified according to *WAPE*. H2 denotes the second forecasting horizon in rolling two-step validation. An asterisk indicates NPR > 0. Such cases are retained for statistical comparison but are excluded from final applicability preference according to the physical-admissibility criterion.

## Data Availability

The original contributions presented in this study are included in the article/Supplementary Materials. Further inquiries can be directed to the corresponding author.

## Supplementary Materials

The following supporting information can be downloaded at: https://www.mdpi.com/article/10.3390/ma19214484/s1, File S1, Supplementary_File_S1_Analysis_Code; Table S1, Supplementary_Table_S1_Complete_Prediction_Outputs.
